# The Cytoplasmic C-Tail of the Mouse Cytomegalovirus 7 Transmembrane Receptor Homologue, M78, Regulates Endocytosis of the Receptor and Modulates Virus Replication in Different Cell Types

**DOI:** 10.1371/journal.pone.0165066

**Published:** 2016-10-19

**Authors:** Nick Davis-Poynter, Joseph Yunis, Helen E. Farrell

**Affiliations:** 1 Child Health Research Centre, The University of Queensland, Brisbane, Australia; 2 School of Chemistry and Molecular Biosciences, The University of Queensland, Brisbane, Australia; University of St Andrews, UNITED KINGDOM

## Abstract

Virus homologues of seven-transmembrane receptors (7TMR) are encoded by all beta- and gammaherpesviruses, suggesting important functional roles. M78 of mouse cytomegalovirus (MCMV) is representative of a family of 7TMR conserved in all betaherpesviruses. M78 family members have been found to exhibit cell-type specific effects upon virus replication in tissue culture and to affect virus pathogenesis *in vivo*. We reported previously that M78, for which no ligands are known, undergoes rapid, constitutive endocytosis. In this study, we have investigated the role of the M78 cytoplasmic C-tail in mediating endocytosis and consequences of C-tail deletion upon replication and pathogenesis. Mutations of M78 (C-tail truncations or point mutations) and CCR5-M78 chimeras identified two distinct regions affecting endocytosis. The first was a classical acidic di-leucine motif (DDxxxLL), located close to the C-terminus. The second region, the activity of which was suppressed by downstream sequences, included the putative 8^th^ helix, located close to the 7^th^ transmembrane domain. A recombinant MCMV expressing an endocytosis-deficient M78, lacking most of the C-tail (M78_CΔ155), had a cell-type specific replication phenotype. M78_CΔ155 had restricted replication in bone marrow macrophages, indistinguishable from an M78-null recombinant. In contrast, M78_CΔ155 replicated normally or with enhanced titres to wild type virus in other tested cell-types, whereas M78-null was attenuated. Distinct phenotypes for M78_CΔ155 and M78-null suggest that the C-tail deletion resulted in M78 dysfunction, rather than complete loss of function; furthermore, they highlight a cell-type specific role of M78 during replication. Infection of mice (intranasal) demonstrated that M78_CΔ155, similar to M78-null, was cleared more rapidly from the lungs than wild type virus and was severely attenuated for replication in salivary glands. It may be speculated that attenuation of both M78_CΔ155 and M78-null for replication in macrophages may have contributed to their similar pathogenic phenotypes.

## Introduction

Seven-transmembrane receptors (7TMR), commonly known as G protein-coupled receptors (GPCR), comprise a large superfamily of membrane proteins that regulate diverse cellular activities. Homologues of 7TMRs have been identified in all beta- and gammaherpesviruses, promoting interest as potential targets for novel antiviral drugs [1, 2]. Phylogenetic analysis suggests six independent gene capture events, three within the betaherpesvirus and three within the gammaherpesvirus lineages [3]. The prototypes of the betaherpesvirus 7TMR gene families were initially identified in human cytomegalovirus (CMV), namely 1) US28 and the related US27, 2) UL33 and 3) UL78 [4]. The US28 gene family is found only in Old World primate CMVs, whereas UL33 and UL78 homologues have been identified in all sequenced betaherpesviruses [5]. Many of the herpesvirus 7TMRs are most closely homologous to chemokine receptors and several have been demonstrated to conserve functions such as chemokine binding and G protein-coupled signalling [1–3].

The UL78 gene family is the least well characterised of the betaherpesvirus 7TMRs. Whereas chemokine binding and signalling has been demonstrated for U51 of both human herpesvirus-6 and 7 (HHV-6/-7) [6, 7], there are no reports of ligand binding or signalling for the CMV UL78 homologues. The majority of reports suggest a conserved role in replication. For mouse and rat CMV, disruption of M/R78 resulted in attenuation of replication in tissue culture and *in vivo* [8, 9]. Similarly, siRNA-mediated knock down of HHV-6 U51 resulted in reduced replication and cell-cell fusion [10]. Whereas disruption of UL78 in a fibroblast-adapted strain of HCMV had little effect upon replication [11], studies using strains that retain broad cell tropism demonstrated a significant effect of UL78 deletion upon replication in certain cell types, in particular retinal pigment epithelial cells [12].

Many cellular 7TMRs have a predominant cell-surface distribution, with endocytosis and recycling triggered following ligand binding. In contrast, constitutive endocytosis has been demonstrated for several viral 7TMRs. Human CMV US28 was shown to localise predominantly to intracellular vesicles, with constitutive endocytosis and recycling in the absence of ligand [13]. Subsequent studies demonstrated constitutive endocytosis of human CMV US27 [14, 15], mouse CMV M78 [16] and human CMV UL78 [17]. It has been hypothesised that endocytosis may recruit these receptors to sites of virion assembly, with implications for virus replication and 7TMR incorporation into the virion envelope. It has also been demonstrated for US28 that constitutive endocytosis depletes extracellular chemokines, similar to the activity of cellular ‘scavenger’ receptors, such as D6 [18, 19].

Endocytosis and trafficking to recycling or lysosomal pathways is a key component of 7TMR regulation, predominantly controlled via interactions between endocytic pathways and the 7TMR cytoplasmic C-tail [20]. A major pathway involves phosphorylation of cytoplasmic residues (via a family of GPCR kinases termed GRKs) and recruitment of β-arrestin. β-arrestin association with the phosphorylated C-tail mediates receptor internalisation via interaction with mediators of endocytosis such as clathrin [21, 22]. The 7TMR is then targeted to either recycling or lysosomal endosomes. An alternative pathway utilised by some cellular 7TMR, which may potentially be regulated by palmitoylation of the cytoplasmic C-tail, involves recruitment of the receptor to lipid rafts where endocytosis may be induced via caveolae-associated or other lipid raft pathways [23, 24]. A motif involved in regulation of endocytosis for a variety of transmembrane proteins, including certain 7TMR, is an acidic dileucine motif ([DE]xxxL[LI]), which is recognised by adaptor-proteins and hence mediates interaction with clathrin [25]. By deletion and C-terminus swap mutagenesis, the C-terminus of US28 was shown to be necessary for endocytosis and sufficient to direct ligand-independent endocytosis of otherwise cell-surface localised 7TMRs [26]. Phosphorylation was required, mediated in part by GRKs, but endocytosis was not dependent on β-arrestins [27–29]. US28 was found to associate with lipid rafts and the C-tail was palmitoylated, but mutation of targets of palmitoylation did not block endocytosis; however, a mutation of a C-terminus proximal acidic dileucine motif (_301_ElhcLL_306_: LL—AA) caused a significant decrease in the endocytosis rate determined via agonist internalisation [27]. A similar motif in the US27 C-tail (_357_EeeeLL_362_) has been suggested to be required for efficient US27 endocytosis [15]. There is evidence that M78 may be internalised via clathrin mediated endocytosis and via lipid rafts/caveolae in transfected cells [16]. However, motifs that drive endocytosis of M78 are yet to be determined.

The hypotheses of this study were that constitutive endocytosis of M78 is mediated via the cytoplasmic C-tail and that endocytosis is essential for M78 function. We have used truncated and chimeric M78 receptor constructs to demonstrate that the M78 C-tail directs rapid, constitutive endocytosis. Two regions of the C-tail are able to induce endocytosis: a region close to the C-terminus which includes an acidic di-leucine motif (_458_DDvsaLL_464_) and a second region overlapping the putative 8^th^ helix (including aa 333–347), the activity of which was unmasked in truncated C-tail constructs. A recombinant MCMV was constructed which expressed an endocytosis deficient M78 mutant lacking most of the C-tail. This recombinant was attenuated for replication similarly to M78 null virus in primary macrophages, but replicated with normal or enhanced titres compared with wild type virus in other cell types tested. In mice infected intranasally, the C-tail deletion mutant displayed a similar phenotype to M78 null virus; whereas replication early post-infection was similar to wild type, the C-tail deletion mutant was cleared more rapidly from the lungs and was severely attenuated for replication in salivary glands.

## Materials and Methods

### Ethics statement

Animal experiments were approved by the University of Queensland Animal Ethics Committee, in accordance with the Australian Animal Care and Protection Act (2001) and the Australian Code for the Care and Use of Animals for Scientific Purposes.

### Virus

The K181 (Perth) strain of MCMV was the ‘wild type’ strain; recombinant viruses derived from K181 were generated via co-transfection/homologous recombination according to previously published methods [30, 31]. Details of the recombinant constructs are given in S1 Table.

### Cell culture

HeLa human epithelial (CCL-2), SVEC 4–10 mouse endothelial (CRL-2181) and NMuMG mouse epithelial (CRL-1636) cell lines originated from the American Type Tissue Culture Collection. Primary mouse embryonic fibroblasts (MEFs—derived from 15–17 day old embryos from outbred ARC(s) mice) and bone marrow macrophages (BMM–derived from adult BALB/c mice) were prepared in-house. HeLa and MEF were maintained in minimal essential medium (MEM, Invitrogen); SVEC and NMuMG were maintained in Dulbecco’s modified Eagle’s medium (DMEM, Invitrogen); BMM were maintained in RPMI-1640 (Invitrogen). Media was routinely supplemented with 10% fetal calf serum (FCS), 2mM glutamine, 100 U penicillin ml^-1^ and 0.1 mg streptomycin ml^-1^ (Invitrogen); BMM were additionally supplemented with 10ng/ml macrophage colony stimulating factor 1 (CSF-1, Peprotech)

### PCR mutagenesis and plasmid construction

An Expand Long template PCR kit (Roche) was used for amplification of truncated/mutated M78 amplicons, which were then cartridge purified (High Pure PCR product purification kit, Roche) and digested. The oligonucleotides used for mutagenesis (SIGMA) and cloning strategies are detailed in S1 Table. Restriction digests used enzymes from New England Biolabs (NEB); plasmid vector DNA was treated with calf intestinal phosphatase (NEB) to prevent self-ligation. Digested products were run on agarose gels and extracted (cartridge purified) prior to ligation (T4 DNA ligase, NEB) and transformation (DH5α C2987, NEB). Transformants were screened by restriction digest and confirmed by sequencing (Australian Genome Research Facility), prior to preparation of DNA from selected clones for transfection (Nucleobond Xtra Midi, Machery Nagel).

### SDS-PAGE and immunoblotting

HeLa cells (2x10^5^ cells/well, 12 well plates) were grown overnight prior to transfection (Lipofectamine 2000). 24 hrs post-transfection, cells were lysed by addition of 150 μl/well RIPA buffer supplemented with HALT protease cocktail (Pierce) and incubated for 10mins at 4°C prior to sonication, centrifugation (8,000 x g, 10mins) and storage of supernatant. Lysate samples (80 μl) were mixed with 30 μl 4xLDS (Invitrogen) and 10 μl 10xDTT (Invitrogen), heated to 50°C for 10 mins then loaded (25 μl) and analysed using NOVEX 4–12% BIS-Tris gel electrophoresis (Invitrogen). Following transfer onto nitrocellulose, filters were blocked with Odyssey blocking buffer (Li-Cor) for 1 hr at room temp, then incubated with 1/1,000 mouse mAb anti-HA (16B12, Covance) in blocking buffer/0.1% Tween-20 (BT) overnight at 4°C. Filters were washed x4 in TBST ((TBS: 50mM Tris, 150mM NaCl, pH = 7.9) /0.1% Tween-20), then incubated with 1/10,000 IRDye-680LT goat anti-mouse (Li-Cor) in BT (1hr, room temp) prior to washing x4 in TBST. Filters were then viewed on an Odyssey scanner (Li-Cor).

### Endocytosis assay

HeLa cells were seeded (10^5^ cells/well using 24 well plates, Corning) on coverslips and grown overnight (37°C, 5% CO_2_) prior to transfection (Lipofectamine 2000, Invitrogen). 24 hours post transfection, cells were incubated with 1/500 Rabbit anti-HA (ab9110, AbCam) in 250μl/well binding medium (OptiMem/0.2% Bovine Serum Albumin (BSA), Invitrogen) supplemented with 1% normal goat serum (NGS, Thermo Scientific). The cells were incubated for 1 hour at 37°C to allow endocytosis, washed twice with pre-warmed OptiMem and incubated for a further 20 minutes at 37°C with binding medium. Cells were then blocked for 15 minutes at 4°C with pre-chilled HBA (Hanks balanced salt solution supplemented with Mg^2+^ & Ca^2+^ (HBSS)/0.2% BSA, Invitrogen) supplemented with 1% NGS, prior to incubation (1hr, 4°C) with 1/1000 AlexaFluor(AF)^594^-Goat anti-rabbit IgG (A11037, Invitrogen) in HBA/0.2% NGS to detect cell surface HA tagged constructs. Cells were then washed 3 times with pre-chilled HBSS, fixed with 3% paraformaldehyde/HBSS (room temp, 20 mins), washed once with HBSS and permeablised with 0.2% Triton X-100 in HBA (room temp, 5 mins). Cells were then washed twice with HBA, blocked with HBA/2% NGS (room temp, 15 mins) prior to incubation (1hr, room temp) with 1/1000 AF^488^ conjugated Goat anti-rabbit IgG (A11034, Invitrogen) in HBA/0.2% NGS. We found that this second anti-rabbit staining was able to detect both internalised and surface localised primary antibody, presumably because the first step anti-rabbit staining did not saturate all available binding sites of the anti-HA primary antibody that was retained at the cell surface. Cells were washed 3 times with HBSS prior to mounting with Prolong Gold/DAPI (Invitrogen). Cells were then viewed under immunofluorescence, either via confocal (Leica TCS SP2) or conventional (NIKON E600) microscopy.

### Image processing and analysis

Endocytosis was quantified using immunofluorescence images from several random fields (NIKON E600, x40 objective), adjusted to minimise background and analysed in ImageJ by selecting regions bounding individual cells to measure signal intensity of the 594 (before permeabilisation) and 488 (after permeabilisation) channels. The endocytosis index was determined as the ratio of signal detected 488/594.

### Virus replication studies

Cells were seeded (24 well plates, medium depending on cell type) and incubated overnight to attain 90% confluence. Approximate cell seeding densities per well: MEF, 2x10^5^; SVEC, 1.5 x10^5^; NMuMG, 1 x10^5^; BMM, 2 x10^5^. Monolayers (triplicate) were then infected (200μl/well) with a multiplicity of infection of 0.01 plaque forming units (pfu)/cell. Following adsorption (1hr, 37°C with periodic rocking), cells were washed twice with medium, replenished with 1ml/well medium (reduced to 2% FCS for MEF, SVEC and NMuMG) and incubated (37°C, 5% CO_2_). At various times post-infection, 200 μl samples of culture fluid were stored (-80°C) and replaced with fresh medium. Virus titres were determined by plaque assay on MEFs.

### Mouse infections

Female BALB/c mice (Animal Resources Centre, Western Australia) were maintained at the University of Queensland Herston Medical Research Centre and used when 6 weeks old. Anaesthesia was achieved by isoflurane inhalation. Viruses were administered intranasally (5 x 10^6^ PFU in 30 μl) to anaesthetized mice. Mice were euthanased by exposure to a rising concentration of CO_2_. Organs were dissected, chilled at 4°C, homogenized, and aliquots were stored at -80°C prior to quantification of infectious virus by plaque assay on MEF.

### Statistical analysis

Data were analysed using Graph Pad Prism 6. Endocytosis indices were log_10_ transformed and statistical significance determined using the Kruskal-Wallis H-test with Dunn’s post-test. Virus titres (log_10_) were analysed using 2-way ANOVA with Bonferroni post-test.

## Results and Discussion

### Identification of regions of the C-tail that direct M78 endocytosis

A plasmid for expression of wild type M78 with an N-terminal HA epitope tag, described previously [16], was used as the template for truncation and substitution mutants of the cytoplasmic C-tail, generated via PCR using the primers listed in S1 Table. Endocytosis was studied in transfected cells using antibody feeding, followed by sequential staining via immunofluorescence of cells pre- and post- permeabilisation, using secondary antibodies conjugated to either AF^594^ (pre-permeabilisation–detection of surface retained M78 only) or AF^488^ (post-permeabilisation–detection of internalised M78 and some surface retained M78)

#### C-terminal truncation mutants

**Fig 1:**
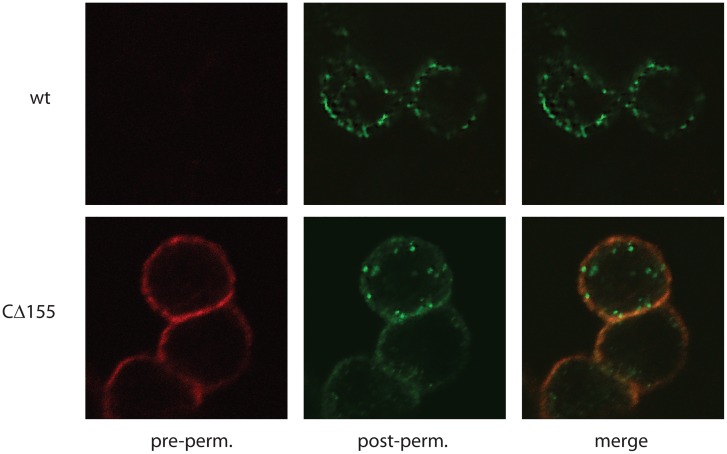
The M78 cytoplasmic C-tail is required for rapid, constitutive endocytosis. Hela cells were transfected with plasmids expressing HA-tagged M78, either wild type (wt) or C-terminally truncated (CΔ155). 1 day post-transfection, cells were fed primary rabbit anti-HA antibody (1hr, 37degs) then stained with secondary anti-rabbit IgG either before (pre-perm. AF^594^ conjugate) or after (post-perm. AF^488^ conjugate) permeabilisation. Confocal images are shown for AF^594^ (red), AF^488^ (green) or the merged channels.

Preliminary studies demonstrated that the M78 C-tail was required for efficient endocytosis, since a mutant deleted of 155 amino acid residues from the C-tail (M78_CΔ155: aa 1–316) retained substantial surface staining, compared with full length M78 (wtM78: aa 1–471), where surface staining was mostly absent (Fig 1 and S1 Fig). However, internalisation of M78_CΔ155 was not completely blocked, since dual staining demonstrated a minor proportion of M78_CΔ155 that was internalised in some cells (Fig 1).

**Fig 2:**
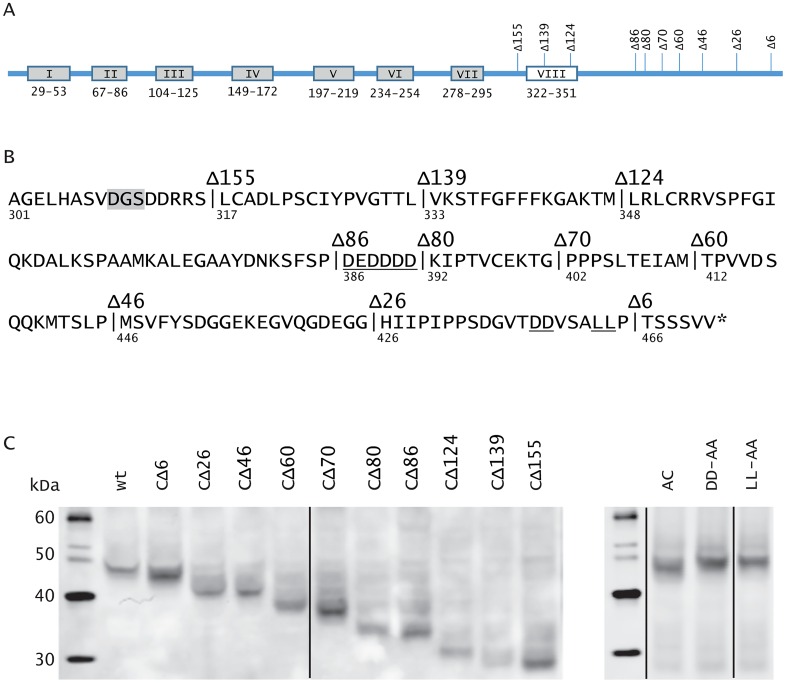
Western blot detection of mutated M78 constructs. Panel A shows a schematic depiction of M78 with boxes indicating positions of the seven predicted transmembrane domains (grey) and putative 8^th^ membrane-associated helix (white); positions of the various truncation mutants are also shown. Secondary structure predictions made via the PredictProtein server [32]. Panel B shows the M78 C-tail amino acid sequence, numbering beneath indicates amino acid position. Vertical lines, with their designations above, indicate positions of truncations. Positions of amino acid substitutions are underlined, namely of the acidic cluster (DEDDDD) and the acidic di-leucine (DDvsaLL). The position corresponding to the SalI site used for certain plasmid constructs is boxed in grey. Panel C shows immunoblot analysis of the various constructs. Hela cells were transfected with plasmids expressing HA-tagged M78, either wild type (wt), C-terminally truncated (CΔ6—CΔ155) or amino acid substitutions disrupting the acidic cluster _386_DEDDDD_391_ (AC: VQNAAA) or the acidic dileucine motif _458_DDxxxLL_464_ (DD-AA or LL-AA). One day post-transfection, cells were harvested and analysed by western blotting, using a mouse monoclonal anti-HA antibody. The marker band sizes are shown (in kDa). The truncation and substitution mutants were analysed on different gels; vertical black lines indicate where the gel images were spliced to remove irrelevant lanes.

**Fig 3:**
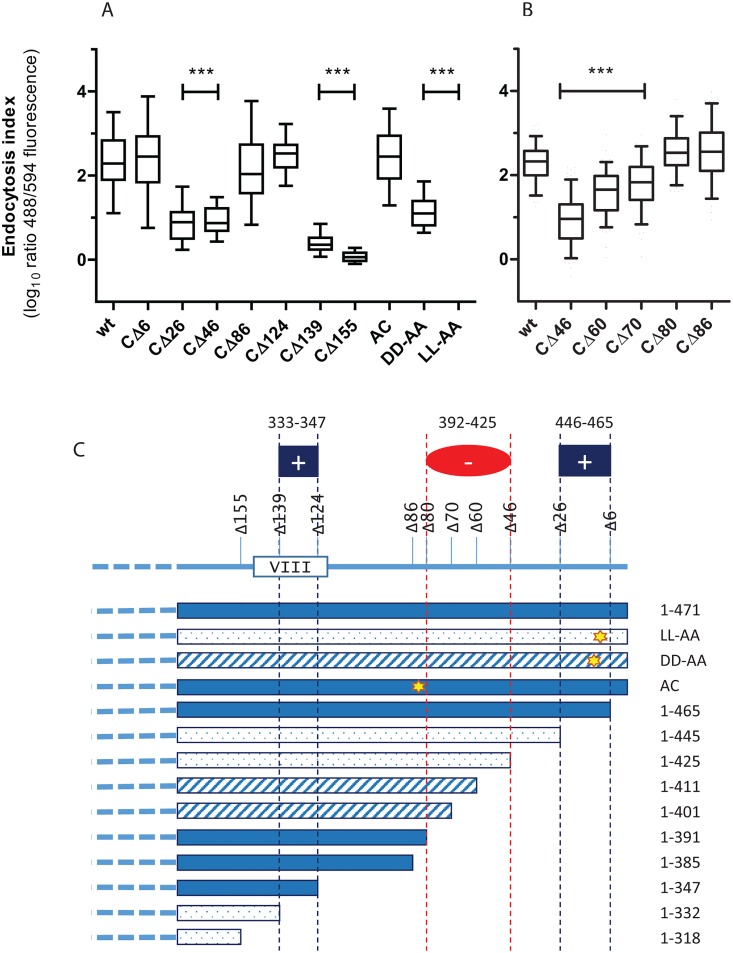
Endocytosis assay of M78 mutants. Hela cells were transfected with plasmids expressing HA-tagged M78, either wild type (wt), C-terminally truncated (CΔ6—CΔ155) or with mutations of the acidic cluster (AC) or the acidic dileucine motif (DD-AA or LL-AA). One day post-transfection, cells were analysed for endocytosis via antibody feeding (rabbit anti-HA, 1hr), then fixed and processed for immunofluorescence either before (AF^594^ conjugated anti-rabbit) or after (AF^488^ conjugated anti-rabbit) permeabilisation. Panels A and B show results for two independent experiments. Coverslips were mounted and images of random fields captured using the 594 and 488 fluorescence filters. Images were analysed using ImageJ software to determine the fluorescence intensity of both the 594 and 488 channels for each transfected cell (>50 cells analysed for each group). The endocytosis index (EI) is plotted as the ratio of intensities (488/594), expressed as log_10_ values. Box and whiskers plots of the endocytosis index are shown, with bars indicating the group median and whiskers the 5^th^ and 95^th^ percentiles. Asterisks indicate statistical significance (Kruskal-Wallis, with Dunn’s post-test) comparing mutants with wild type M78 (***P<0.001). The various constructs and effects upon endocytosis are depicted schematically in Panel C. Solid shading indicates a median EI >2, hatched shading indicates 2>EI>1, and stippled shading EI<1.

A panel of C-tail truncation mutants was analysed to identify regions of the M78 C-tail modulating endocytosis, using transient transfection of HeLa cells. Western blotting confirmed expression of each of the constructs (Fig 2). Quantification of endocytosis was achieved by antibody feeding and sequential immunofluorescence staining to determine an endocytosis index, expressed as the ratio of signal for the two different secondary antibody fluorescence channels AF^488^/AF^594^, corresponding to detection of internalised (plus some surface)/surface M78. A ratio of approx. 1 (0 on log_10_ scale) indicates cell-surface retention whereas higher ratios indicate endocytosis. Results (Fig 3A) suggested that two different regions of the C-tail were capable of inducing endocytosis. The putative regulatory regions and effects of M78 mutation upon endocytosis are depicted schematically in Fig 3C. A region close to the C-terminus (aa 446–465) was suggested as a positive regulator of endocytosis, since M78_CΔ6 (aa 1–465) behaved similarly to wtM78, whereas M78_CΔ26 (aa 1–445) had substantially reduced endocytosis (*P*<0.001). A second positive regulator was suggested (aa 333–347), potentially within the putative 8^th^ membrane-associated helix, since M78_CΔ139 (aa 1–332) was deficient for endocytosis (*P*<0.001) whereas M78_CΔ124 (aa 1–347) was efficiently endocytosed. These results also suggested that a region downstream of aa 385 may inhibit endocytosis induced by the upstream element. This was probed by an additional set of truncation mutants (CΔ80, CΔ70, CΔ60), with results shown in Fig 3B. A region between aa 392–425 was suggested a negative regulator, since whereas M78_CΔ80 (aa 1–391) had an endocytosis index similar to wtM78, M78_CΔ46 (aa 1–425) was poorly endocytosed. M78_CΔ60 (aa 1–411) and M78_CΔ70 (aa 1–401) had an intermediate phenotype, with enhanced endocytosis compared with M78_CΔ46 (*P*<0.001), but reduced endocytosis compared with M78_CΔ80 (*P*<0.001). This suggests either that the region between aa 392–425 includes motifs that have a specific inhibitory effect upon the upstream endocytosis signal, or alternatively that there is non-specific inhibition related to the length of the C-tail.

#### Mutation of potential sorting motifs

Two motifs apparent in the C-tail that may potentially influence endocytosis were mutated (results shown Fig 3A & 3C). A known endocytic signal, namely an acidic di-leucine motif (_458_DDvsaLL_464_), was disrupted by alanine substitution of either the acidic residues (DD-AA) or the leucines (LL-AA). Both mutations resulted in significant inhibition of endocytosis (*P*<0.001). Clusters of acidic residues, with accompanying serine residues, have been identified as modulators of endocytosis/trafficking in some proteins, including the protease furin, the neurotransmitter transporter VMAT-2 and the human CMV envelope glycoprotein B [33–35]. An acidic cluster apparent in the M78 C-tail (AC: _386_DEDDDD_391_) was modified by multiple substitutions (VQNAAA) with no apparent effect on endocytosis efficiency. Taken together, the truncation and motif mutant results suggested that the di-leucine motif close to the C-terminus was the dominant signal for endocytosis, with a secondary ‘cryptic’ signal including the region between aa 333–347, which is masked in the presence of a downstream region between aa 392–425.

### The M78 C-tail has motifs that can direct constitutive endocytosis of an unrelated 7TMR

To determine whether the M78 C-tail is sufficient to induce endocytosis of an unrelated 7TMR and to further delineate positive and negative regulatory regions, we constructed a series of CCR5/M78 C-tail chimeras, using an approach similar to those reported previously for human CMV US28 and US27 [15, 26]. The full length CCR5 coding sequence (HA-CCR5: aa 1–352), or a truncated CCR5 lacking the cytoplasmic C-tail and with a XhoI site incorporated for cloning purposes (HA-CCR5trunc: aa 1–305), were amplified by PCR (S1 Table) and introduced into an expression vector downstream of a self-cleaving signal peptide and HA-tag. CCR5/M78 chimeras were generated by insertion of M78 C-tail regions from selected mutant constructs (digested with SalI sites present within the M78 coding sequence and downstream of the stop codon) at the XhoI site of HA-CCR5trunc. The various constructs were then analysed for endocytosis as described previously.

**Fig 4:**
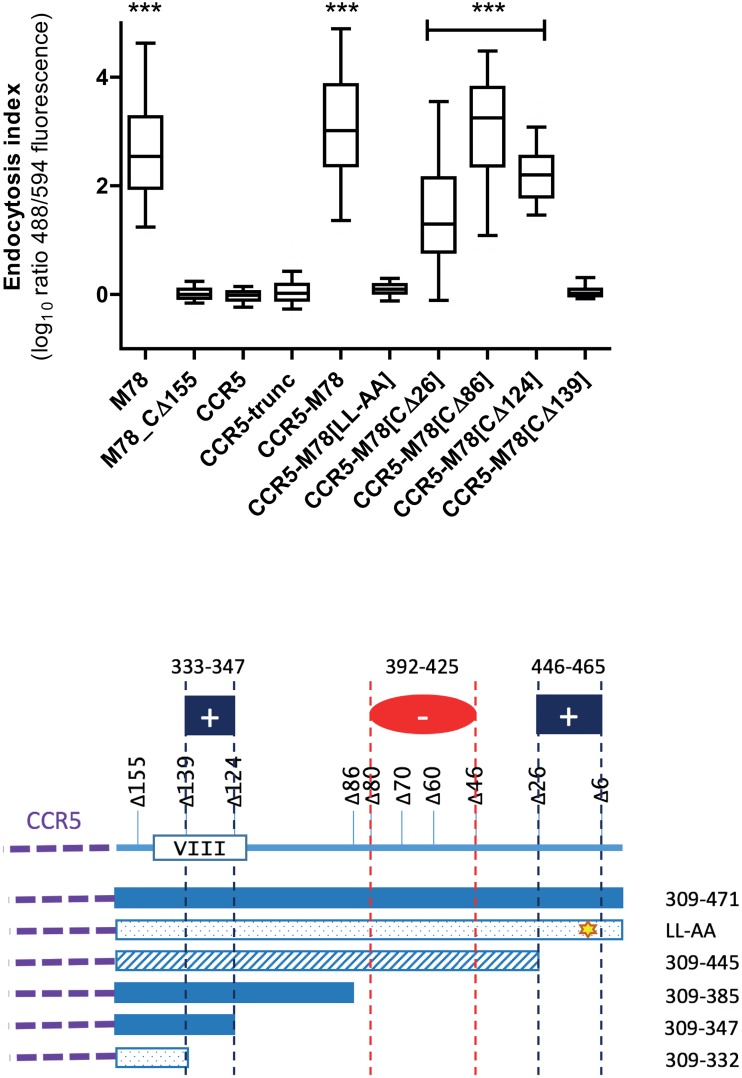
Endocytosis assay of CCR5/M78 chimeras. Hela cells were transfected with plasmids expressing HA-tagged CCR5, either wild type (CCR5), truncated to remove the C-tail (CCR5 trunc), or chimeric with the C-tail replaced by M78 C-tail sequences either wild type (CCR5-M78) or mutated (LL-AA, CΔ26 –CΔ139). HA-M78 and HA-M78_CΔ155 were included as controls. One day post-transfection, cells were analysed for endocytosis as described for Fig 3. Box and whiskers plots of the endocytosis index (EI) are shown, with bars indicating the group median and whiskers the 5^th^ and 95^th^ percentiles. Asterisks indicate statistical significance (Kruskal-Wallis, with Dunn’s post-test) comparing CCR5-trunc with the other constructs. (***P<0.001). The various chimeric constructs and effects upon endocytosis are depicted schematically in Panel B. Solid shading indicates a median EI >2, hatched shading indicates 2>EI>1, and stippled shading EI<1.

As expected, HA-CCR5 and HA-CCR5trunc were retained mostly at the cell surface (Fig 4A). Addition of the full length M78 C-tail (aa 309–471: HA-CCR5/M78) resulted in efficient endocytosis, similar to HA-M78, demonstrating that the M78 C-tail is sufficient to direct constitutive endocytosis (Fig 4A). The putative regulatory regions and effects of mutation upon endocytosis are depicted schematically in Fig 4B. The full length C-tail with disruption of the di-leucine motif (HA-CCR5/M78[LL-AA]) was deficient for endocytosis, confirming this motif as the dominant signal for endocytosis (Fig 4A). However, consistent with the data from the M78 constructs, a second region of the M78 C-tail may direct endocytosis in truncation mutants lacking the di-leucine motif. Thus, HA-CCR5/M78[CΔ124] (aa 309–347) was endocytosed with an endocytosis index > 2 (log_10_), whereas [CΔ139] (aa 309–332) was retained at the cell surface (Fig 4A & 4B). These data support the hypothesis that the region between aa 333–347 includes a secondary ‘cryptic’ signal for endocytosis. It is tempting to speculate that the putative 8^th^ helix of M78 (aa 322–351, Fig 2) is a major component of the secondary endocytosis signal. In support for this proposal, the putative 8^th^ helix of D6 was implicated in regulation of D6 endocytosis, since disruption (by truncation) of the 8^th^ helix resulted in defective scavenging of CCL3 [36].

### The M78 C-tail is not required for efficient replication in most cell types

**Fig 5:**
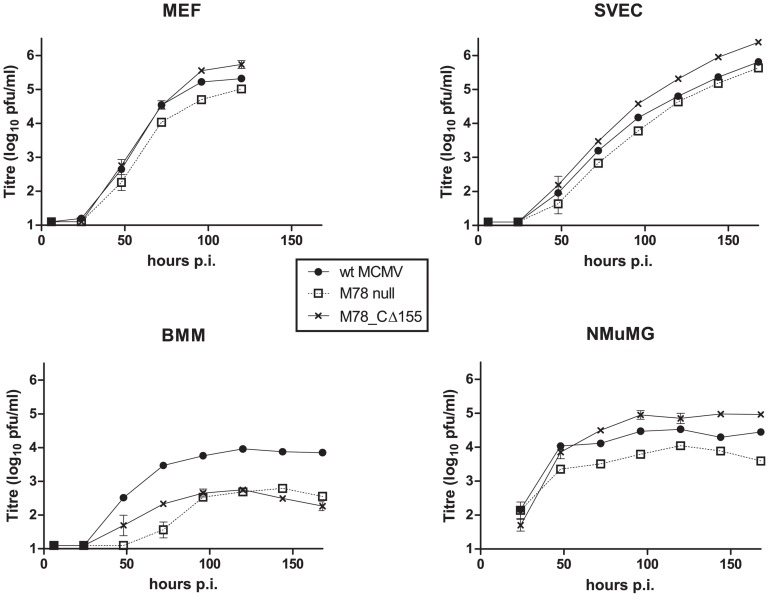
Effects of M78 mutation upon replication kinetics in different cell types. Multi-step growth curves of wild type (wt), M78_null and M78_ CΔ155 in fibroblast (MEF), endothelial (SVEC), epithelial (NMuMG) and macrophage (BMM) cell types. Cells were infected in 24 well trays at low multiplicity (0.01 pfu/cell) and supernatant harvested at various times post-infection for titration of infectious virus by plaque assay (mean and standard error shown; n = 3). Statistical significance was determined via 2-way ANOVA with Bonferroni post-test and is reported in the text.

To determine whether constitutive endocytosis of M78 is functionally important for virus replication, we constructed a recombinant MCMV expressing M78_CΔ155. This was generated by first disrupting M78 by insertion of a lacZ selectable marker (recombinant M78 null), then using M78 null as the parent for generation of recombinant M78_CΔ155, whereby the M78_CΔ155 sequence replaced the M78/lacZ sequence by homologous recombination, using methods similar to those employed for M33 mutation [30] (further details of plasmid constructs for generation of recombinants is given in S1 Table. Recombinant viruses were plaque purified and verified by PCR/sequencing. Multi-step growth curves were determined in a number of different cell types, namely mouse fibroblast (MEF), endothelial (SVEC), epithelial (NMuMG) and bone marrow-derived macrophage (BMM), with results shown in Fig 5. Preliminary studies of transiently transfected cells confirmed that the CΔ155 truncation inhibited endocytosis compared to wild type M78 in MEF, SVEC and NMuMG cells (S2 Fig); the effect in BMM could not be assessed due to inefficient transfection. Previous studies of an M78 null mutant (Smith strain, EGFP insertion) reported a moderate replication defect (≤ 10-fold) following low multiplicity infection in fibroblasts (10.1 –embryonic fibroblast derived cell line) and a more pronounced attenuation (approx. 50 fold) in macrophages (IC21 –peritoneal macrophage-derived cell line) [9]. The results for the M78 null virus of this study are similar, with marked attenuation in BMM (10–80 fold reduced titre; *p*<0.001 from 48–168 hours post-infection) and lesser attenuation in the other cell types (SVEC, up to 3 fold; MEF, up to 4 fold; NMuMG, up to 7 fold). The M78_CΔ155 mutation attenuated replication in BMM (5–40 fold reduced titre; *p*<0.001 from 48–168 hours post-infection), with replication kinetics similar to that of M78 null. In contrast, M78_CΔ155 replicated at least as well as wild type MCMV in the other cell types tested, with moderately higher titres in SVEC (3–4 fold; *p*<0.01 at 120–168 hours post infection) and NMuMG (3–5 fold; *p*<0.05 at 96, 144 and 168 hours post infection). The above results suggest that the M78_CΔ155 mutation resulted in dysregulation of M78 function, with phenotypic effects distinct from knock-out of M78.

### The M78 C-tail is required for replication in salivary glands following intranasal infection

**Fig 6:**
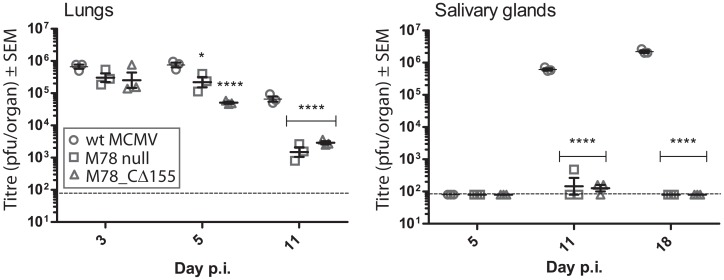
Effects of M78 mutation upon replication *in vivo*. Mice were infected intranasally under anaesthesia and organs harvested at different days post-infection. Infectious virus titres recovered from lungs and salivary glands are shown as individual data points (n = 3 mice per group) with the mean and standard error of the mean indicated by bars. Asterisks indicate statistical significance comparing wt with the mutant viruses (2-way ANOVA with Bonferroni post-test): * *P*<0.05; ****P<0.0001.

Replication of the M78 null and CΔ155 recombinants was compared with wt MCMV *in vivo*, following intranasal inoculation (Fig 6). In the lungs, the recombinants replicated to similar titres as wt MCMV initially (day 3 p.i.), but were cleared more rapidly (approximately 20 -to 40-fold lower titres than wt MCMV by 11 days post-infection (*P*<0.0001)). In salivary glands, both recombinants were severely attenuated (over 1000-fold lower titres than wt MCMV at days 11 and 18 post-infection (*P*<0.0001)). These results are consistent with deletion of the M78 C-tail, with concomitant disruption of M78 endocytosis, resulting in dysregulation of M78 function in cell types relevant to clearance from the lung and replication in salivary glands. Given the attenuation of the C-tail deletion mutant in macrophages, but not other cell types tested *in vitro*, we speculate that a role of M78 during infection of macrophages may contribute to the observed *in vivo* phenotypes.

## Conclusion

Consistent with the hypothesis that the cytoplasmic C-tail of M78 directs endocytosis, two regions were identified that induced rapid endocytosis of M78, namely an acidic di-leucine (_458_DDxxxLL_464_) and another cryptic signal (aa 333–347), which lies within the putative 8^th^ helix of M78, but has no obvious homology to known endocytic motifs. Studies of CCR5/M78 chimeras confirmed the activity of these C-tail regions was not dependent on properties of the body of the receptor. Furthermore, the chimera studies showed activity in the absence of CCR5 ligands, supporting the hypothesis that M78 endocytosis is constitutive, rather than mediated by a ubiquitous (as yet unidentified) ligand. Contrary to the hypothesis that endocytosis is essential for M78 function, an MCMV expressing an endocytosis deficient M78 mutant (M78_CΔ155), lacking most of the C-tail, was not equivalent to M78_null. With the exception of bone marrow macrophages (BMM), M78_CΔ155 had normal or enhanced replication in a variety of cell types, whereas M78_null was attenuated. The dysfunction of M78_CΔ155 resulted in a profound attenuation *in vivo* following intranasal infection, similar to that of M78_null. Given the known contribution of monocyte/macrophage infection to the pathogenesis of MCMV [37], it is tempting to speculate that defective macrophage replication contributed to the phenotypes observed for M78_CΔ155 and M78_null. Further studies are warranted to define the functions of M78 and related proteins of other betaherpesviruses, to explore their potential as an anti-viral therapeutic target.

## Supporting Information

S1 Table Oligonucleotides used for plasmid constructs and mutagenesis. (DOCX)

S1 Fig Preliminary characterisation of M78 endocytosis. (EPS)

S2 Fig Preliminary characterisation of M78 endocytosis. (EPS)
