# Supplementary material for: The Cytoplasmic C-Tail of the Mouse Cytomegalovirus 7 Transmembrane Receptor Homologue, M78, Regulates Endocytosis of the Receptor and Modulates Virus Replication in Different Cell Types
Source: PLoS One. 2016 Oct 19;11(10):e0165066. doi: 10.1371/journal.pone.0165066 (PMC5070858; doi:10.1371/journal.pone.0165066)
Supplement: S1 Table — (DOCX) [file pone.0165066.s001.docx]

# S1 Table

**Oligonucleotides used for plasmid constructs and mutagenesis**

| M78_EcoHAf | CGGAATTCAAGCCATGCCGACTTCATC |
| --- | --- |
| M78_stopERIr | CGGAATTCAGACAACAGAGGAGGAGGTAGG |
|  |  |
| M78_CΔ6r | CGGAATTCAAGGCAGTAGGGCCGAGAC |
| M78_CΔ26r | CGGAATTCATCCTCCTTCATCGCCCTGC |
| M78_CΔ46r | CGGAATTCAGGGTAGGGACGTCATCTTCTG |
| M78_CΔ60r | CGGAATTCACATGGCGATCTCGGTG |
| M78_CΔ70r | CGGAATTCATCCGGTCTTCTCACAGACGG |
| M78_CΔ80r | CGGAATTCAGTCGTCGTCGTCCTCG |
| M78_CΔ86r | CGGAATTCAGGGCGAGAAACTCTTGTTGTC |
| M78_CΔ124r | CCGGAATTCACAGCATCGTCTTGGCGCCC |
| M78_CΔ139r | CGGAATTCAGAGGGTGGTTCCGACCGGG |
| M78_CΔ155r | CGGAATTCACAAAGACCGGCGGTCATCGG |
| M78_ACf | GTGCAGAACGCGGCCGCGAAGATCCCCACCGTCTGTGAG |
| M78_ACr | CGCGGCCGCGTTCTGCACGGGCGAGAAACTCTTGTTGTC |
|  |  |
| M78_LL-AAf | CTCGGCCGCCGCCCCTACCTCCTCCTC |
| M78_LL-AAr | GGAGGTAGGGGCGGCGGCCGAGACATC |
| M78_DD-AAf | GTGACCGCGGCGGTCTCGGCCCTACTG |
| M78_DD-Aar | CGAGACCGCCGCGGTCACCCCGTCGGA |
|  |  |
| CCR5_BamHAf | CGGCGGATCCATGGATTATCAAGTGTCAAG |
| CCR5_stopBHIr | CGGCGGATCCGTGTCACAAGCCCACA |
|  |  |
| CCR5tr_Xhor | CGGCGCTCGAGTCTGAACTTCTCCCCGACA |

## HA-M78 expression

The construct for expression of HA-tagged M78 [16], was generated by PCR amplification of the M78 coding sequence from MCMV genomic DNA (K181-Perth) using primers M78_EcoHAf and M78_stopERIr, which was inserted at the EcoRI site of pCMV-HA (Clontech). Derivatives were generated via PCR amplification of M78 truncated sequences or point mutations, which were similarly cloned into pCMV-HA via EcoRI sites.

## HA-CCR5 and HA-CCR5/M78 expression

The HA tagged expression vector pcDNA_SIG-HA was generated by oligonucleotide insertion between the HindIII and BamHI sites (underlined) of pcDNA3.1 (Invitrogen) as shown below. The self-cleaving signal peptide is shown in italics and the HA epitope tag is shown in bold.

pcDNA3.1_SIG-HA

AAGCTTGCCACCATGAAGACGATCATCGCCCTGAGCTACATCTTCTGCCTGGTATTCGCCGGATCTTACCCATACGATGTTCCAGATTACGCTGGATCC

*M K T I I A L S Y I F C L V F A* G S **Y P Y D V P D Y A** G S

HA-tagged CCR5 constructs were generated by PCR amplification of full length or truncated CCR5, using primers CCR5_BamHAf and either CCR5_stopBHIr or CCR5tr_Xhor respectively. The amplicons were digested with BamHI (full length) or BamHI and XhoI (truncated) and cloned into pcDNA_SIG-HA, via the BamHI or BamHI/XhoI sites, to generate pHA-CCR5full and pHA-CCR5tr respectively. Chimeric HA-CCR5/M78 constructs were generated by insertion of SalI fragments from the various pHA-M78 truncated/mutated constructs at the XhoI site of HA-CCR5tr.

## Recombination vectors

**M78_null**

A region of MCMV genomic DNA (K181-Perth) spanning the M78 locus (corresponding to ntd 107,316 – 115,082; genbank accession AM886412) was inserted as an EcoRI/EcoRV fragment into BluescriptII SK between the EcoRI and SmaI sites to generate pBS-M78. The M78 ORF was disrupted by insertion of a lacZ expression cassette (PstI fragment of pMV11 [31]) at a NsiI site approx.. 600ntds downstream from the M78 start codon, to generate pBS-M78_null.

**M78_CΔ155**

pBS-M78 was modified by PCR to introduce an MfeI site (underlined) incorporating the endogenous stop codon (bold): CAAT**TGA**AA. The resultant pBS-M78_MfeStop was digested with AvrII/MfeI and the corresponding regions of M78 mutants (truncated or point mutation), derived from the expression plasmids, inserted as AvrII/EcoRI fragments.
